# Comparison of the Prevalence of Common Bacterial Pathogens in the Oropharynx and Nasopharynx of Gambian Infants

**DOI:** 10.1371/journal.pone.0075558

**Published:** 2013-09-23

**Authors:** Aderonke Odutola, Martin Antonio, Olumuyiwa Owolabi, Abdoulie Bojang, Ebenezer Foster-Nyarko, Simon Donkor, Ifedayo Adetifa, Sylvia Taylor, Christian Bottomley, Brian Greenwood, Martin Ota

**Affiliations:** 1 Vaccinology Theme, Medical Research Council Unit, Banjul, The Gambia; 2 Global Epidemiology, GlaxoSmithKline Vaccines, Wavre, Belgium; 3 Faculty of Infectious and Tropical Disease, London School of Hygiene & Tropical Medicine, London, United Kingdom; 4 World Health Organization, Regional Office for Africa, Brazzaville, Congo; University of Cambridge, United Kingdom

## Abstract

**Background:**

CRM- based pneumococcal conjugate vaccines generally have little impact on the overall prevalence of pneumococcal carriage because of serotype replacement. In contrast, protein vaccines could substantially reduce the overall prevalence of pneumococcal carriage with potential microbiological and clinical consequences. Therefore, trials of pneumococcal protein vaccines need to evaluate their impact on carriage of other potentially pathogenic bacteria in addition to the pneumococcus.

**Methods:**

As a prelude to a trial of an investigational pneumococcal vaccine containing pneumococcal polysaccharide conjugates and pneumococcal proteins, the prevalence of carriage of *Streptococcus pneumoniae*, *Haemophilus influenzae*, *Moraxella* species and *Staphylococcus aureus* in the nasopharynx of 1030 Gambian infants (median age 35 weeks) was determined. An oropharyngeal swab was obtained at the same time from the first 371 infants enrolled. Standard microbiological techniques were used to evaluate the bacterial flora of the pharynx and to compare that found in the oropharynx and in the nasopharynx.

**Results:**

The overall pneumococcal carriage rate was high. Isolation rates of *S. pneumoniae* and *Moraxella* species were significantly higher using nasopharyngeal rather than oropharyngeal swabs (76.1% [95% CI 73.4%,78.7%] vs. 21.3% [95% CI 17.2%,25.8%] and 48.9% [95% CI 45.8%, 52.0%] vs. 20.5% % [95% CI 16.5%,25.0%] respectively). In contrast, *S. aureus* and *H. influenzae* were isolated more frequently from oropharyngeal than from nasopharyngeal swabs (65.0% [95% CI 59.9%, 69.8%] vs. 33.6% [95% CI 30.7%, 36.5%] and 31.8% [95% CI 16.5%, 25.0%] vs. 22.4% [95% CI 19.9%, 25.1%] respectively). No group A β haemolytic *streptococci* were isolated.

**Conclusion:**

Collection of an oropharyngeal swab in addition to a nasopharyngeal swab will provide little additional information on the impact of a novel pneumococcal vaccine on pneumococcal carriage but it might provide additional, valuable information on the impact of the vaccine on the overall microbiota of the pharynx.

## Introduction

Pneumonia remains one of the leading causes of mortality in children < 5 years old [1]. In 2010, it was responsible for 18% of the approximately 7.6 million deaths in children in this age group, with approximately 50% of these deaths occurring in sub-Saharan Africa [2]. *Streptococcus pneumoniae* (the pneumococcus) accounts for 30-50% of pneumonia-related deaths and is a leading cause of death in children < 2 years of age in developing countries [3]. Other bacterial pathogens that frequently cause severe acute infections in childhood include *Haemophilus influenzae*, *Neisseria* *meningitidis* and *Staphylococcus aureus*. These pathogens, together with many commensal bacteria, constitute the bacterial microbiome of the pharynx [4]. There is increasing interest in the potential for interaction between the many bacterial species resident in the pharynx and in the possibility that interventions targeted at one species, or against a restricted group within a species, may influence the overall bacterial flora. For example, at the species level there is evidence for a negative interaction between *S. aureus* and *S. pneumoniae* in the nasopharynx [5-8] and competition within species is shown by the phenomenon of serotype replacement following pneumococcal conjugate vaccination [9]. For these reasons, consideration needs to be given to the possibility that introduction of a new vaccine targeted at one bacterial species resident in the pharynx could have an impact on the overall bacterial flora of the pharynx, with potential clinical consequences.

A trial of an investigational pneumococcal vaccine containing 10 pneumococcal polysaccharide non-typeable *H. influenzae* conjugates and two pneumococcal proteins proteins (PhtD and dPly) [10] is being undertaken in Gambian infants (ClinicalTrials.gov NCT01262872). As a prelude to this trial, a study was undertaken in the same area where the vaccine trial is being conducted of the prevalence of pneumococcal carriage in Gambian infants who had received two or three doses of a seven-valent pneumococcal conjugate vaccine (PCV-7) as part of their routine immunisation schedule to determine the prevalence in infants of carriage with pneumococci of non vaccine type (NVT) that might be affected by the protein vaccine. This preliminary study has also provided an opportunity to determine whether collection of an additional oropharyngeal (OP) as well as a nasopharyngeal (NP) swab would help in establishing the impact of this investigational vaccine on the overall bacterial flora of the pharynx.

## Subjects and Methods

### Study area and population

The study was conducted in Fajikunda, a peri-urban area situated on the western margin of The Gambia, approximately 20 km from the capital Banjul. Fajikunda, with a population of approximately 50,000, is typical of communities on the margins of large West African cities. The population includes members from all the main ethnic groups in The Gambia and also residents from other countries in West Africa. The population is engaged in a wide range of occupations, including working for government services, professions and trading. The climate of the area is typical of the sub-Sahel with a long dry season from December to July and a shorter rainy season with an average annual rainfall of around 1200 mm. The study described in this paper was conducted at the end of the dry season.

### Survey methods

Prior to the start of the study, the Fajikunda community was informed of its purpose and the nature of the investigations to be performed, starting with community and religious leaders followed by the general population. The Fajikunda study area was then mapped and a census taken of all households. Pregnant women and children under the age of 1 year in each household were identified and their date of birth and immunization record were documented. Entry criteria for the study were an age of 12 months or less and previous immunisation with two or three doses of a seven-valent pneumococcal conjugate vaccine (PCV-7) (Prevenar^R^), with the last dose having been given at least one month before sampling (At the time of the study PCV-7 was part of the Gambian national infant immunisation programme but it has recently been replaced by a 13-valent vaccine). Households were sampled systematically across the study area and no geographical selection, for example of households with a higher than average social status, was made. Infants who had received three doses of PCV-7 were recruited first and then those who had received two doses, with the last dose having been given at least one month before sampling, in order to achieve the required sample size.

The study was conducted between April and July 2010. Field workers trained in the procedure of obtaining OP and NP swabs visited study subjects in the community. A history of immunizations, breastfeeding and recent antibiotic use was obtained. Coverage with three doses of pentavalent vaccine in the study area was over 90%. Weight was recorded and the infant’s immunization card examined to obtain the dates on which each dose of PCV-7 had been administered. A calcium alginate-tipped swab was then inserted gently through the nostril into the NP, left to soak for about 5 seconds with gentle rotation, and removed. An OP specimen was obtained using the same type of swab passed through the mouth to the posterior wall of the pharynx. After samples had been obtained, the tip of each of the ultrafine applicator swabs was cut off and placed into a tube containing skim milk-tryptone-glucose-glycerol (STGG) transport media, the tube placed in a cold box with ice-packs, transported within eight hours to the MRC unit at Fajara, and then held frozen at -70°C until analysed. Previous studies conducted in The Gambia [11] and elsewhere [12] have shown that storage of STGG samples at -70°C for even prolonged periods does not decrease the viability of pharyngeal bacteria.

### Microbiological methods

Following thawing of the inoculated STGG sample to room temperature, each tube of NP- or OP-STGG was vortexed vigorously for a minimum of 20 seconds to homogenise the bacterial suspension. The suspension was then plated out on Blood Agar (BA), Gentamicin supplemented blood agar (GBA), Mannitol salt agar (MSA), Chocolate agar (CA) and Bacitracin supplemented chocolate agar (BCA) plates for the isolation of Group A *streptococci*, *S. pneumoniae*, *S.* aureus*,* *Moraxella* *species* *and H. influenzae* respectively. All plates were incubated at 37°C overnight; MSA and BA in O_2_, GBA, BCA and CA in 5% CO_2_.

Characterisation of the five species of bacteria commonly found in the oropharynx or nasopharynx was carried out based on their characteristic morphology, Gram stain and biochemical properties as follows:

#### S. pneumoniae

Small, grey, moist/mucoidal colonies showing alpha-haemolysis on a blood agar plate were selected for catalase and optochin testing. Catalase negative, optochin sensitive colonies were serotyped by latex agglutination using serotype specific antisera (Statens Serum Institute, Copenhagen, Denmark) as described previously [13]. Confirmation of serotype was made by PCR in an approximately 20% of randomly selected samples [14] and PCR was also used to differentiate between serotypes 6A and 6C.

#### H. influenzae

Following overnight incubation, large, round, colourless to grey opaque colonies were selected from the BCA plate for oxidase testing and exposure to X+V factors. Isolates which were oxidase positive and X+V growth factor dependent were confirmed as *H. influenzae*. These were serotyped using BD Difco^TM^ Hi antisera type a-f (BD, Oxford Science Park, Oxford, UK).

#### S. aureus

Yellowish, slightly raised, colonies on MSA were sub-cultured onto BA plates to obtain pure colonies. Catalase and Remel-Staphaurex® (coagulase) (Oxoid & Remel Products, Thermo, Fisher Scientific, Basingstoke, Hants, UK) positive isolates were confirmed as *S. aureus.*

#### *Moraxella* *species*

Grey-white, dry and brittle colonies easily lifted with a wire loop were sub-cultured from the CA for further testing. Oxidase positive, DNase positive, Gram negative cocci, dipolococci or coccobacilli were confirmed as *Moraxella catarrhalis*.

#### Group A β-haemolytic streptococci

Colourless, dry, shiny or mucoid colonies showing beta haemolytic colonies were selected for catalase and bacitracin testing. Suspected isolates were typed with Remel Streptex kit (Oxoid & Remel Products, Thermo, Fisher Scientific, Basingstoke, Hants, UK) to confirm that they were Group A streptococci (GAS).

The MRC Unit, submits to the external quality assurance programme of the United Kingdom National External Quality Assessment Service [15] and is a World Health Organization (WHO) Regional Reference Laboratory for invasive bacterial pathogens.

### Data entry and analysis

Data forms were double entered, their validity checked and queries resolved.

The prevalence of *S. pneumoniae*, *S.* aureus*, H. influenzae, M. catarrhalis* and the Group A β- haemolytic streptococcus in OP and NP samples was determined. The serotype distribution of OP and NP isolates of *S. pneumoniae* was compared. A p-value for this comparison was obtained by non-parametric bootstrap to allow for the dependence between samples (individuals positive for *S. pneumoniae* on both swabs contributed data to both samples).

The sensitivity of OP and NP swabs was compared for each bacterial species in individuals from whom both OP and NP swabs were taken. Individuals positive on either swab were considered carriers of the bacterium, i.e. true positives. The kappa statistic [16] was used to assess the level of agreement between OP and NP swabs.

Pairwise odds ratios were calculated to quantify the strength of the association between the bacterial species. The odds ratios were estimated using the Mantel-Haenszel method to adjust for age in months, sex and for ethnic group (Madinka, Jola, Fula, Wollof, Sarahule and others).

Risk factors for carriage of *S. pneumoniae* in the nasopharynx were identified using logistic regression. Odds ratios for the effects of antibiotic use, breastfeeding and number of PCV doses were adjusted for age, which was modelled as a fractional polynomial of degree two, ethnicity and gender.

The study was approved by the Gambia Government/Medical Research Council Unit Joint Ethics Committee and by the ethics committee of the London School of Hygiene & Tropical Medicine. Individual written consent was taken from the parents or guardians of all study infants before investigation.

## Results

### Characteristics of the study population

Nasopharyngeal swabs were collected from 1031 children from a potentially eligible population of 1487 infants. The parents of 31 infants declined the invitation to join the study, the families of 47 infants could not be found at the time of the survey and the families of 378 were not approached after the pre-set sample size had been reached. The characteristics of the infants enrolled in the trial (ethnicity, age, sex, weight, breastfeeding experience, recent antibiotic use and number of doses of PCV-7 received) are shown in Table 1. OP swabs were collected from the first 371 subjects studied (financial constraints precluded the collection of a larger number of OP samples). There were no significant differences between the characteristics of children from whom only an NP swab was obtained and those from whom both OP and NP swabs were collected (Table 1).

**Table 1:** Characteristics of study population.

		OP+NP N (%)	NP only N (%)	All N (%)
Ethnicity	Mandinka	156 (42)	203 (31)	359 (35)
	Jola	74 (20)	123 (19)	197 (19)
	Fula	55 (15)	118 (18)	173 (17)
	Wollof	48 (13)	101 (15)	149 (14)
	Sarahule	14 (4)	41 (6)	55 (5)
	Other	24 (6)	73 (11)	97 (9)
	Total	371 (100)	659 (100)	1030 (100)
Gender	Male	211 (57)	359 (54)	570 (55)
	Female	160 (43)	301 (46)	461 (45)
	Total	371 (100)	660 (100)	1031 (100)
Antibiotic use since birth	No	340 (92)	611 (93)	951 (92)
	Yes	31 (8)	49 (7)	80 (8)
	Total	371 (100)	660 (100)	1031 (100)
Feeding practice^*^	None	1(0)	4 (1)	5(0)
	Mixed feeding	331 (89)	570 (86)	901 (87)
	Breast milk + water	21 (6)	63 (10)	84 (8)
	Breast milk only	18 (5)	23 (3)	41 (4)
	Total	371 (100)	660 (100)	1031 (100)
No. doses PCV	2	35 (9)	101 (15)	136 (13)
	3	336 (91)	559 (85)	895 (87)
	Total	371 (100)	660 (100)	1031 (100)
Age in weeks	Median (IQR)	35.3(27.4,46.9)	35.4(27.1,46.6)	35.3(27.4,46.6)
Weight (Kg)	Median (IQR)	8.1(7.0,9.0)	7.6(6.7,8.5)	7.7(6.8,8.7)

* Feeding practice at the time that a swab was collected.

### Nasopharyngeal swabs

**Figure 1:**
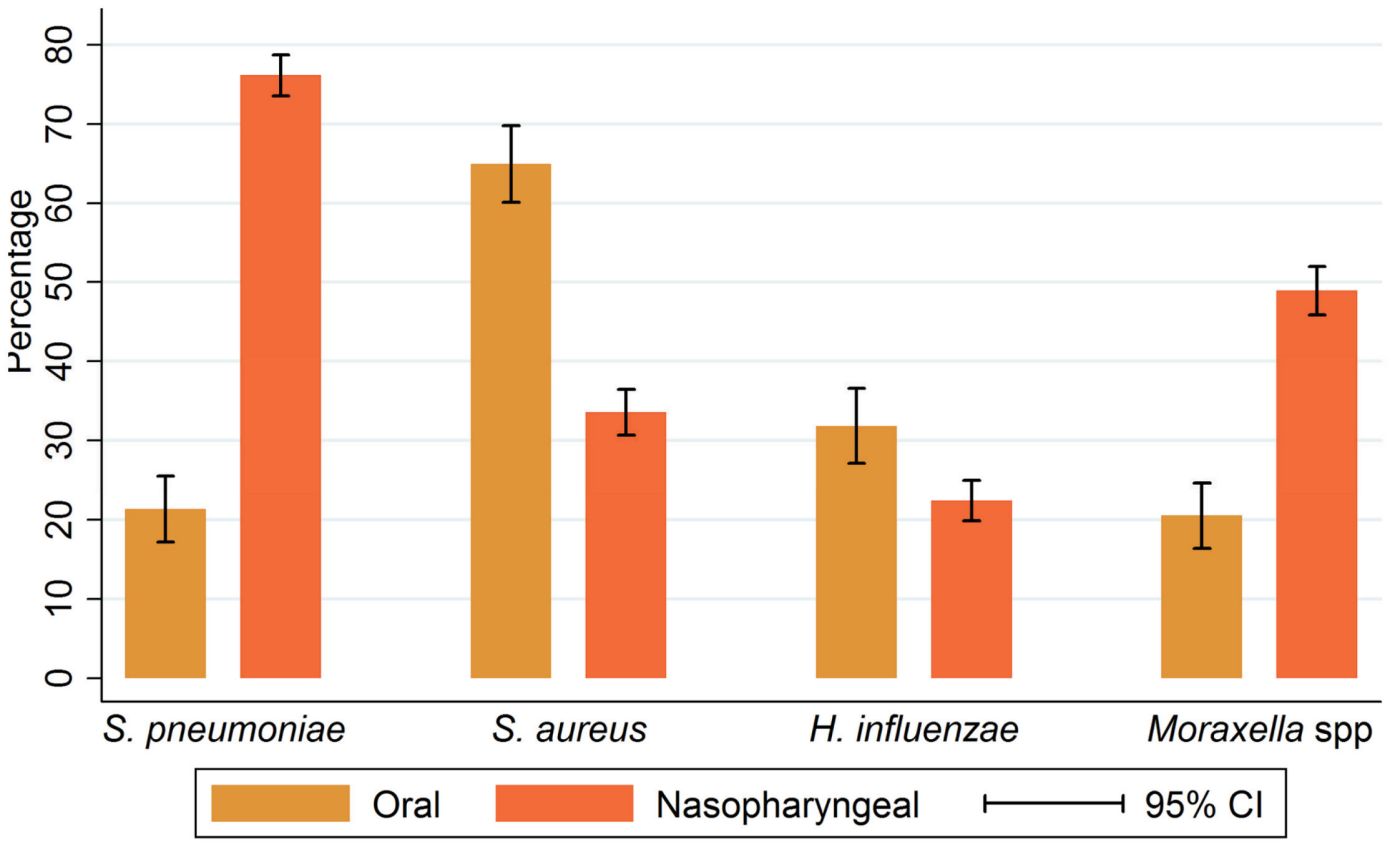
Prevalence (%) of bacterial species obtained from oral or nasopharyngeal swabs.

**Figure 2:**
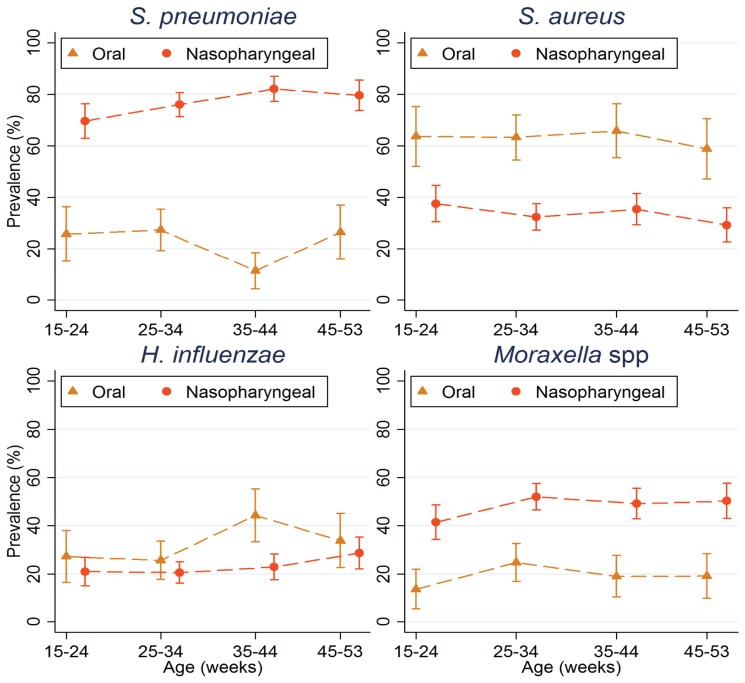
Age distribution by week of age of pathogens by site of isolation. Circles indicate nasopharyngeal swabs, triangles oropharyngeal swabs.

*S. pneumoniae* was the commonest pathogen isolated from NP swabs (76.1% [95% CI 73.4%, 78.7%] followed by *M. catarrhalis* (48.9%) [95% CI 45.8%, 52.0%) (Figure 1). *S. aureus* was isolated from 33.6% [95% CI 30.7%, 36.5%] of NP swabs and *H. influenzae* from 22.4% [95% CI 19.9%, 25.1%]. No group A β-haemolytic *streptococci* were isolated from NP swabs. The prevalence of NP carriage by age for the four study organisms identified is shown in Figure 2. The prevalence of carriage of *S. pneumoniae*, *H. influenzae* and *Moraxella* species increased gradually with age, whilst the prevalence of carriage with *S. aureus* showed a trend in the opposite direction.

The serotype distribution of the pneumococci obtained from NP swabs is shown in Table 2. The commonest serotype carried in the NP was 6A. The serotypes covered by PCV-7 constituted 14.5% of the pneumococcal isolates, while 15.3% and 38.0% belonged to serotypes covered by PCV-10 and PCV-13 respectively. No serotype 6C isolate was detected. The prevalence of PCV-7 vaccine serotypes and non-vaccine serotypes was similar in infants who had received three or two doses of PCV-7 [11.2% vs. 9.6%; OR = 1.16 (0.62, 2.17) and 65.5% vs. 67.6%, OR = 0.84 (0.57, 1.26) respectively]. One hundred and sixty-eight *H. influenzae* isolates could be reactivated for serotyping; 13 (7.7%) belonged to serotype b, 9 (5.4%) to serotype a, 10 (6.0%) to serotype c, 2 (1.2%) to serotype d, 4 (2.4%) to serotype e, 3 (1.8%) to serotypes f, whilst the remaining 127 (75.5%) were untypeable.

**Table 2:** Prevalence of vaccine serotypes among swabs positive for *S. pneumoniae*.

	Oropharyngeal % (95% CI) (N=79)	Nasopharyngeal % (95% CI) (N=785)	P-value
1	0.0(0.0,4.6)	0.0(0.0,0.5)	NA
3	8.9(3.6,17.4)	2.5(1.6,3.9)	0.052
4	0.0(0.0,4.6)	0.4(0.1,1.1)	NA
5	1.3(0.0,6.9)	0.5(0.1,1.3)	0.539
6A	11.4(5.3,20.5)	14.6(12.2,17.3)	0.367
6B	5.1(1.4,12.5)	0.9(0.4,1.8)	0.096
7F	0.0(0.0,4.6)	0.3(0.0,0.9)	NA
9V	0.0(0.0,4.6)	0.0(0.0,0.5)	NA
14	7.5(2.8,15.6)	4.7(3.3,6.4)	0.349
18C	0.0(0.0,4.6)	0.3(0.0,0.9)	NA
19A	5.1(1.4,12.5)	5.6(4.1,7.5)	0.831
19F	6.3(2.1,14.2)	6.0(4.4,7.9)	0.899
23F	5.1(1.4,12.5)	2.2(1.3,3.4)	0.215
PCV7^1^	23.8(14.9,34.6)	14.4(12.0,17.0)	0.048
PCV10^2^	25.0(16.0,35.9)	15.2(12.7,17.9)	0.052
PCV13^3^	47.5(36.2,59.0)	37.7(34.3,41.2)	0.084

NA = Not Applicable

1 PCV7 serotypes include serotypes 4, 6B, 9V, 14, 18C, 19F, and 23F plus 6A which shows cross- protective immunity with serotype 6B.

2 PCV10 serotypes include the above plus serotypes 1, 5, 7F

3 PCV-13 serotypes include the PCV7 serotypes plus 1, 3, 5, 6A, 7F, 19A

Interactions between the different species of bacteria in the nasopharynx are shown in Table 3. A statistically significant positive association was found between the presence of *S. pneumoniae* and *H. influenzae* [OR = 1.86 (1.21, 2.87)]. No significant association between the presence of *S. pneumoniae* and *S. aureus* was observed [OR= 1.05 (0.74, 1.47)]. Approximately 8% of infants were reported to have received antibiotics since birth. No significant difference was seen in the prevalence of carriage of *S. pneumoniae, H. influenzae, M. catarrhalis or S. aureus* between the 80 infants whose parents or guardians reported the administration of antibiotics than in the 951 infants without a history of recent antibiotic use.

**Table 3:** Odds ratios with 95% confidence intervals (adjusted for age, sex and ethnicity) for the associations between bacterial species found in the oropharynx or nasopharynx.

**Nasopharynx**				
	*S. pneumoniae*	*S. aureus*	*H* *. influenza*	*M. catarrhalis*
*S. pneumoniae*	1	1.05(0.74,1.47)	1.86(1.21,2.87)	1.15(0.83,1.60)
*S. aureus*		1	1.39(0.99,1.95)	1.22(0.92,1.62)
*H. influenzae*			1	1.36(0.99,1.87)
*M. catarrhalis*				1
**Oropharynx**				
	*S. pneumoniae*	*S. aureus*	*H* *. influenza*	*M. catarrhalis*
*S. pneumoniae*	1	0.82(0.46,1.46)	1.29(0.71,2.33)	1.25(0.62,2.52)
*S. aureus*		1	1.65(0.95,2.85)	1.46(0.80,2.69)
*H. influenzae*			1	2.11(1.12,3.96)
*M. catarrhalis*				1

### Oropharyngeal swabs

The bacteria isolated from OP swabs are shown in Figure 1. The commonest pathogen isolated from the OP was *S. aureus* (65.0% [95% CI 59.9%, 69.8%]) followed by *H. influenzae* (31.8% [95% CI 27.1%, 36.8%])*. S. pneumoniae and M. catarrhalis* were isolated from 21.3% [95% CI 17.2%, 25.8%], 20.5% [95% CI 16.5%, 25.0%] of OP swabs respectively. No serogroup A β-haemolytic *streptococci* were obtained. The distribution of potential pathogens in the OP by age is shown in Figure 2. This was similar to the pattern seen for NP swabs.

The pneumococcal serotype distribution in the OP was generally similar to that of the NP, with serotype 6A (11.4%) being found most frequently (Table 2). However, the prevalence of the serotypes covered by the PCVs was generally higher in the OP than in the NP swabs, being 23.8%, 25.0% and 47.5% for PCV-7, PCV-10 and PCV-13 respectively in the NP isolates compared to 14.4%, 15.2% and 37.7% among the OP isolates, with differences for several serotypes being of borderline statistical significance (Table 2). Seventeen *H. influenzae* isolates could be reactivated for serotyping; 1 belonged to serotype b, 1 to serotype c, and 1 to serotype d, whilst the remaining 14 (82.4%) were untypeable.

A positive association was found between carriage of *H. influenzae and M. catarrhalis* [OR = 2.11 (1.12, 3.96)] and between carriage of *H. influenzae* and *S. aureus* [OR= 1.65 (0.95, 2.85)]. No significant association between carriage of *S. pneumoniae* and *S. aureus* was observed.

### Comparison of the findings from oral and nasopharyngeal swabs

Assuming that the detection of a bacterial species in either the NP or the OP as indicative of pharyngeal colonisation, the sensitivity of OP and NP swabs at detecting specific species are shown in Table 4. These estimates are likely to over-estimate sensitivity since some colonisation will have been missed by both swabs using conventional microbiological as opposed to more sensitive molecular techniques. Nasopharyngeal swabs were significantly more sensitive than OP swabs at detecting *S. pneumoniae* and *M. catarrhalis* whilst the reverse was the case for *S. aureus* and *H. influenzae*. The kappa statistics gave low values for the comparisons between OP and NP swabs for all four species studied.

**Table 4:** Sensitivity of oral and nasopharyngeal swabs for detecting four bacterial species in the pharynx.

	N†	Oropharyngeal % (95% CI)	Nasopharyngeal % (95% CI)	Kappa (95% CI)
*S. pneumoniae*	278	28.4(23.2,34.1)	95.7(92.6,97.7)	0.09(0.03,0.15)
*S. aureus*	266	90.6(86.4,93.8)	32.0(26.4,37.9)	0.04(-0.03,0.11)
*H. influenzae*	170	69.4(61.9,76.2)	46.5(38.8,54.3)	0.03(-0.07,0.12)
*M. catarrhalis*	214	35.5(29.1,42.3)	81.8(75.9,86.7)	0.01(-0.07,0.10)

Kappa represents the level of agreement.

† Number of individuals carrying the bacteria on at least one of the swabs. Oral and nasopharyngeal swabs were obtained from 371 individuals.

### Risk factors for carriage

The prevalence of carriage in the nasopharynx increased significantly with age from 15 weeks up to 1 year (Table 5), and was highest amongst Fula and Jola children. Carriage was reduced among children who had been treated with antibiotics at some point since birth and among children who had received three, rather than two, doses of pneumococcal conjugate vaccine; although neither of these latter two associations was statistically significant.

**Table 5:** Risk factors for carriage of *S. pneumoniae* in the nasopharynx.

		N	Carriage %	OR†	Pvalue
Age (weeks)	15-24	181	69.6	1	
	25-34	321	76.0	1.38(0.92,2.08)	
	35-44	240	82.1	2.00(1.27,3.16)	
	45-53	181	79.6	1.70(1.05,2.75)	0.020
Ethnicity	Mandinka	359	70.8	1	
	Jola	197	83.2	2.05(1.33,3.18)	
	Fula	173	83.8	2.14(1.35,3.41)	
	Wollof	149	77.2	1.40(0.90,2.18)	
	Sarahule	55	67.3	0.85(0.46,1.56)	
	Other	97	72.2	1.07(0.65,1.77)	0.001
Gender	Male	570	75.8	1	
	Female	461	76.6	1.04(0.78,1.39)	0.769
Antibiotic use since birth	No	951	76.7	1	
	Yes	80	70.0	0.72(0.43,1.20)	0.210
Breastfeeding	None or mixed	906	76.0	1.00	
	Exclusive or with water	125	76.8	1.24(0.77,2.00)	0.384
PCV No. doses	2	136	76.5	1	
	3	895	76.1	0.89(0.57,1.39)	0.620

† Odds ratios are unadjusted except for antibiotic use, breastfeeding and number of PCV doses which are adjusted for age, ethnicity and gender. Age adjusted for using fractional polynomials of degree 2.

## Discussion

This study was conducted to determine the carriage rates of *S. pneumoniae*, *S.* aureus, *H. influenzae* and *Moraxella* species in infants who had been vaccinated with PCV-7 prior to the future conduct of a trial of an investigational pneumococcal vaccine in a similar group of infants resident in the same area. The study was undertaken in a representative sample of a peri-urban area near Banjul, the capital of The Gambia, and its findings are likely to reflect those that would be found in the large peri-urban areas of other West African cities where PCVs have been introduced. A very high pneumococcal carriage rate (76.1% [95% CI 73.4%, 78.7%]) was found, as recorded in The Gambia on several previous occasions before the introduction of PCV-7 regardless of whether the survey was conducted in the dry or rainy season [13,17,18,19]. In these earlier studies, 40%-50% of pneumococcal isolates belonged to serotypes covered by PCV-7. However, following vaccination with PCV-7 or with an experimental 9-valent conjugate vaccine, the proportion of isolates of PCV-7 serotype fell to around 10%, a figure similar to that observed in this study (14.5%) [19,20]. The high prevalence of carriage of pneumococci of non-PCV10 and PCV-13 vaccine serotypes in the nasopharynx of infants in the trial community (65% and 47% respectively) will facilitate the ability of the trial of the investigational pneumococcal vaccine to determine whether it has any impact on carriage of serotypes of pneumococci not covered by the PCV-10 or PCV-13 conjugate vaccines with which it is being compared.

There was little influence of age on carriage of any of the potential pathogens studied during the first year of life, although there was a trend towards increasing prevalence with age of carriage with *S. pneumoniae* and *H. influenzae* but a decrease with age for *S. aureus*, as noted in a previous detailed study in Gambian infants which used molecular diagnostic methods [21].

Isolation rates of four common bacterial colonisers of the pharynx obtained with either a NP or an OP swab were compared. Nasopharyngeal swabs were more sensitive at detecting *S. pneumoniae* and *M. catarrhalis* whereas OP swabs were more sensitive at detecting *S. aureus* and *H. influenzae*. The finding of a higher isolation of pneumococci from NP than OP swabs is in keeping with previous findings in children [22-25] and in adults [26,27] in industrialised countries and with the results of a study undertaken in children in the Philippines [28]. In Israel, the higher isolation rate of pneumococci from NP as opposed to OP was more marked in children and in their mothers [29]. We are unaware of any previous study that has made this comparison in a situation of high intensity pneumococcal transmission such as that found in sub-Saharan Africa. Comparison of isolation rates from NP and OP of bacterial species other than the pneumococcus have been few and have given more varied results. In children in the UK [22], the Philippines [28] and Israel [29] isolation rates of *H. influenzae* were not statistically different when NP or OP swabs were used. However, in children in Finland [24]and adults in Israel [29] the isolation rate of *H. influenzae* was higher using OP rather than NP swabs, as we found in our study. Even fewer studies have looked at differential colonisation rates by *Moraxella* species but, in Israeli adults, these bacteria were isolated more frequently from NP than from OP swabs [27] as was the case in our study.

There is increasing interest in interactions between bacterial species in the nasopharynx because of the possibility of interrupting this balance through vaccination, with possible clinical consequences. In our study, a positive association was found between the presence of *S. pneumoniae* and *H. influenzae* in both the nasopharynx and the oropharynx, as noted in South African children [6] but the association was statistically significant only in the nasopharynx. This association may be due to the fact that colonisation with these two species of common bacteria has common risk factors related to socio-economic status, nutritional status and hygiene. We did not find a negative association between *S. pneumoniae* and *S. aureus*, as had been expected as this has been noted previously in Gambian infants [21] and in studies elsewhere [5,6,7]. The reason why this negative interaction was not apparent is not clear; it might have been hidden by the confounding effect of risk factors common to the two pathogens, but it is unlikely to have been due to seasonality or recent changes in antibiotic practice. Absence of an inverse correlation between the presence of *S. pneumoniae* and *S. aureus* is an encouraging finding as it suggests that if the new protein vaccine does reduce the overall prevalence of pneumococcal carriage this may not lead to a concomitant increase in carriage of *S. aureus.*

Little is known about the reasons for the differences in preferential localisation of potential pathogens in the upper respiratory tract. Preferential selection of sites for colonisation could reflect the presence of different epithelial cell ligands in the oropharynx and nasopharynx or it could be a reflection of differential innate or acquired immune responses at these two sites. Although OP swabs gave a pneumococcal carriage rate about three times less than the NP, the proportion of serotypes covered by PCV7 was higher in the OP than in the NP isolates. This could have been a chance finding but it could also indicate that the induction of local immunity to carriage by PCV-7 is stronger in the NP than in the OP.

A weakness of this study is that, for financial reasons, it was only possible to compare results obtained by OP or NP swabs in a sub-sample of study infants. However, the fact that infants in the OP + NP and NP alone groups were well matched for characteristics that may have influenced carriage rates and the high overall rate of carriage of the various bacteria studied makes the findings of this comparative study robust.

The findings of this study indicate little additional information will be gained by collecting an OP swab in addition to a NP swab if the sole focus of an intervention is to evaluate its impact on carriage of pneumococci. If, however, there is concern that an intervention may have a more general effect on the overall bacterial flora of the pharynx then both OP and NP swabs may be needed.
